# Pretreatment with probiotic *Enterococcus faecium* NCIMB 11181 ameliorates necrotic enteritis-induced intestinal barrier injury in broiler chickens

**DOI:** 10.1038/s41598-019-46578-x

**Published:** 2019-07-16

**Authors:** Yuanyuan Wu, Wenrui Zhen, Yanqiang Geng, Zhong Wang, Yuming Guo

**Affiliations:** State Key Laboratory of Animal Nutrition, College of Animal Science and Technology, China Agricultural University, Beijing, China

**Keywords:** Applied microbiology, Microbiome, Animal physiology

## Abstract

The dysfunction of tight-junction integrity caused by necrotic enteritis (NE) is associated with decreased nutrient absorption and gut injury in broiler chickens. Although probiotic *Enterococcus faecium* (*E. faecium*) has been reported to possess immune-regulatory characteristics and can prevent diarrhea in pigs, very little information exists in relation to the specific regulatory impact of *E. faecium* NCIMB 11181 on NE-induced intestinal barrier injury of broiler chickens. This study was conducted to investigate the protective effects of probiotic *E. faecium* NCIMB 11181 on NE-induced intestinal barrier injury in broiler chickens. The study also aimed to elucidate the mechanisms that underpin these protective effects. One hundred and eighty Arbor Acres (AA) broiler chicks (one day old) were randomly assigned using a 2 × 2 factorial arrangement into two groups fed different levels of dietary *E. faecium* NCIMB 11181 (0 or 2 × 10^8^ CFU/kg of diet) and two disease-challenge groups (control or NE challenged). The results showed that NE induced body weight loss, intestinal lesions, and histopathological inflammation, as well as intestinal-cell apoptosis. These symptoms were alleviated following the administration of probiotic *E. faecium* NCIMB 11181. Pretreatment with probiotic *E. faecium* NCIMB 11181 significantly upregulated the expression of the Claudin-1 gene encoding a tight-junction protein. Claudin-1 and HSP70 protein expression were also increased in the jejunum regardless of NE infection. Furthermore, NE-infected birds fed with *E. faecium* displayed notable increases in MyD88, NF-κB, iNOS, PI3K, GLP-2, IL-1β, IL-4, and HSP70 mRNA expression. *E. faecium* NCIMB 11181 administration also significantly improved the animals’ intestinal microbial composition regardless of NE treatment. These findings indicated that addition of *E. faecium* NCIMB 11181 to poultry feed is effective in mitigating NE-induced gut injury, possibly by strengthening intestinal mucosal barrier function, as well as modulating gut microflora and intestinal mucosal immune responses.

## Introduction

Necrotic enteritis (NE) is caused by *Clostridium perfringens* (*C*. *perfringens*) types A and C. *C*. *perfringens is* a spore-forming, anaerobic, gram-positive, rod-shaped bacterium that produces a range of necrotizing toxins including α-toxin and NetB toxin^1^. NE infection in broiler chickens disrupts the composition of their intestinal microbial community^2–4^, damages gut morphology, causes intestinal inflammation, and impairs gut barrier function^5,6^; these chickens also exhibit increased gut permeability^7,8^ and depressed growth^6^. Thus, strategies to alleviate the detrimental effects of NE infection on intestinal mucosal barrier integrity are of great significance for the health of broiler chickens.

Evidence has indicated that the administration of probiotic bacteria improves intestinal function by maintaining paracellular permeability, enhancing the physical mucous layer, stimulating the immune system, and modulating the composition and activity of resident microbiota^9^. The regulatory effects of probiotics on human, pig, and chicken intestinal homeostasis have been extensively studied^10,11^, and interactions between probiotic bacteria, commensal bacteria, and the epithelial barrier are thought to be important in this regard. *Enterococcus faecium* (*E. faecium*) is one of the most important lactic acid-producing bacterial species belonging to the autochthonous microbiota of human and animal gastrointestinal tracts. The *Enterococcus* genus is also found in the microbiota of multifarious food sources^12,13^, with some species being commonly used as probiotics in food preservatives or feed additives because they produce antimicrobial substances such as organic acids and bacteriocins^14,15^. These species also exert positive effects on disease incidence, do not carry virulence-factor genes, and are sensitive to specific antibiotics^12,13,16^. In animals, *E. faecium* probiotics are mainly used to treat or prevent diarrhea, to facilitate immune stimulation, or to improve growth. For example, feed supplementation with probiotic *E. faecium* has been shown to protect pigs from the pathogenic bacteria *E*. *coli*^17–19^. *E. faecium* also protected against viruses, chlamydia, and parasitic infections in swine and mice^20–22^. Specifically, these protective effects reduced the pathogenic bacterial load in internal organs, impeded virulence-gene expression by resident pathogens, and/or modulated inflammatory responses, thereby reducing the number of piglets suffering from diarrhea and improving their growth performance^17–19,23,24^. Furthermore, studies have shown that the addition of *E. faecium* to sow feed possibly modified the composition of the pigs’ intestinal bacterial community by increasing the prevalence of beneficial bacteria and reducing pathogenic bacterial load^25^. *In vitro* studies have demonstrated that *E. faecium* can affect trans-epithelial electrical resistance and epithelial permeability while also modulating tight-junction (TJ) protein expression and distribution^26–28^. In addition, results from experiments in poultry have revealed that dietary *E. faecium* probiotics modulated intestinal microflora composition^29,30^, restrained the spread of pathogens, stimulated intestinal mucosal immune responses, and enhanced the birds’ resistance to intestinal pathogen infections including those caused by *Salmonella*, *E. coli*, *Campylobacter*, and *C. perfringens*^31–34^.

Probiotic *E*. *faecium* strain 11181 is currently authorized by the European Food Safety Authority (EFSA) Panel as a feed supplement for fattening and improving the growth performance of animals^16,35^. This strain has been shown to effectively increase daily weight gain and improve feed conversion in weaning piglets^36^. Pajarillo *et al*.^35^ also reported that administration of *E*. *faecium* NCIMB 11181 to pigs enhanced gut health by promoting the growth of beneficial bacteria and inhibiting the proliferation of gut pathogens. In addition, our previous study also found that adding *E. faecium* NCIMB 11181 at 5 × 10^7^ CFU/kg to the diet of broiler chickens improved growth performance, while a dietary inclusion of 1–2 × 10^8^ CFU/kg stimulated the systemic immune response of broiler chickens^37^. However, the effects of probiotics on intestinal barrier function are strain-dependent and not ubiquitous. Moreover, very little information exists regarding the regulatory impact of probiotic *E*. *faecium* NCIMB 11181 strains on NE-induced intestinal barrier injury and the associated mechanisms that underpin these reactions. Therefore, the objectives of this study were (1) to test whether oral supplementation with *E*. *faecium* NCIMB 11181 protected gut barrier function against NE infection and (2) to characterize the changes of intestinal microbiome as well as the Toll-like receptor (TLR)/NF-κB signal pathway-mediated mucosal immune response.

## Results

### Growth performance

**Figure 1:**
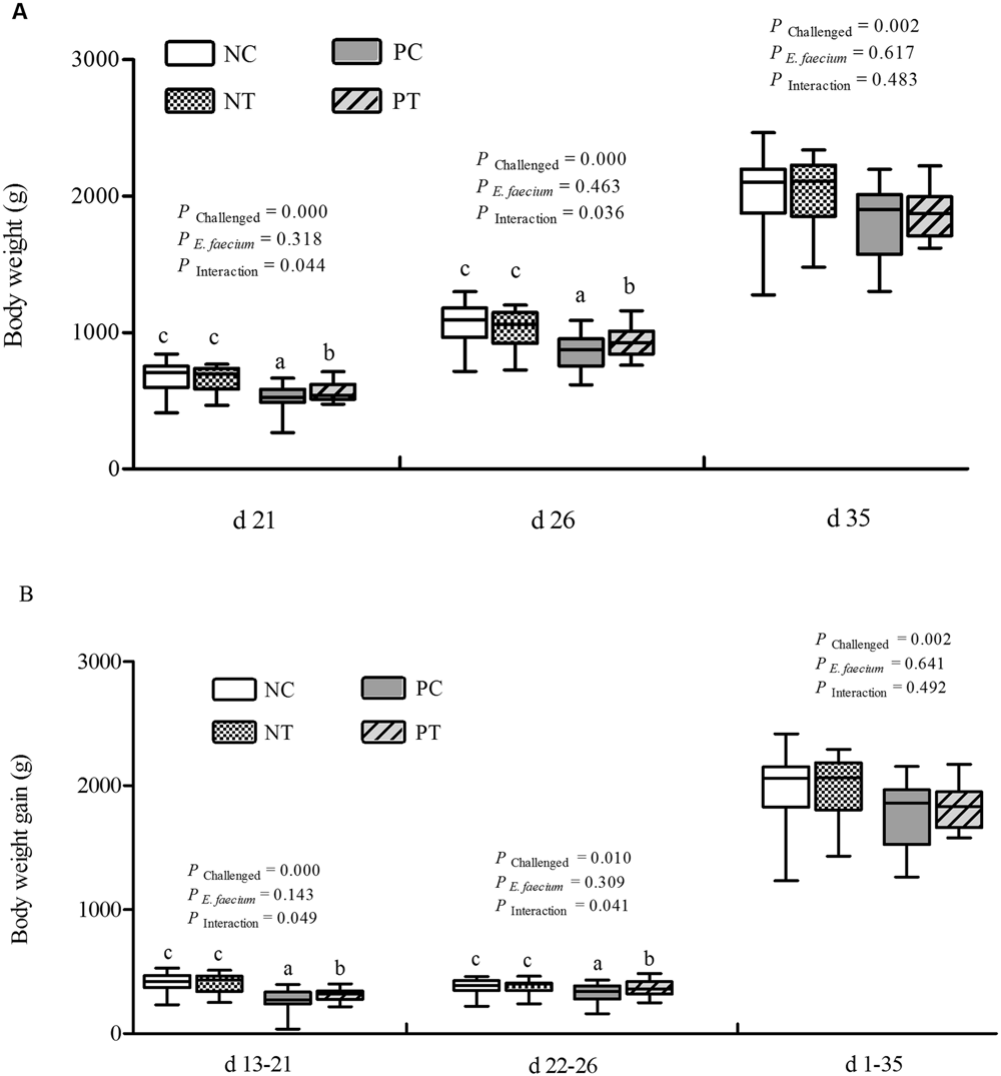
Effect of dietary *E*. *faecium* 11181 on growth performance of broiler chickens challenged with NE. (**A**) Body weight at d 21, 26, and 35 (3, 7, and 15 days post-infection [DPI]). (**B**) Body-weight gain during d 13–21, d 22–26, d 27–35, and d 1–35 (challenge-3 DPI, 3–7 DPI, 7–17 DPI, and the entire testing period). Individual data points are presented as box plots, showing the median (horizontal lines), the lower and upper quartiles (lower and upper borders of the boxes), and minimum and maximum values (lower and upper whiskers). The different lowercase letters on the bars indicate significant differences (*P* < 0.05). NC = Non-NE-infected + no *E. faecium* treatment, NT = non-NE-infected + *E*. *faecium* treatment, PC = NE-infected + no *E*. *faecium* treatment, PT = NE-infected + *E*. *faecium* treatment.

The evaluation of body weight (BW) and body-weight gain (BWG) in broiler chickens in the NE-challenge group is illustrated in Fig. 1. Compared with the unchallenged birds, NE-challenged birds exhibited significant reductions in BW (*P* < 0.01) and BWG during the specific challenge periods as well as the entire testing period (*P* < 0.05). A strong interaction between dietary *E*. *faecium* and NE infection was observed in relation to BW at day (d) 21 and d 26, and BWG at d 13–21 and d 22–26 (*P* < 0.05). NE-challenged birds fed diets supplemented with *E*. *faecium* showed a significant improvement in BW and BWG compared with NE-challenged birds fed only the basal diet (*P* < 0.05).

### Liver bacterial translocation

**Figure 2:**
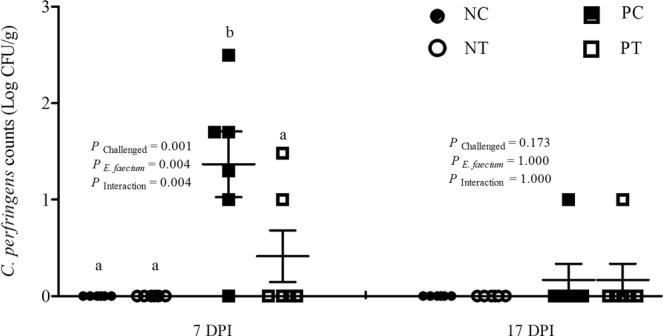
Effect of dietary *E*. *faecium* 11181 on bacterial translocation to the liver of broiler chickens challenged with NE. Each dot (n = 6) represents an individual sample under different treatments. The lowercase letters on the dots indicate significant differences (*P* < 0.05). NC = Non-NE-infected + no *E. faecium* treatment, NT = non-NE-infected + *E. faecium* treatment, PC = NE-infected + no *E. faecium* treatment, PT = NE-infected + *E. faecium* treatment.

A significant cooperative effect (*P* < 0.05) was found between *E*. *faecium* addition and NE infection in terms of *C*. *perfringens* invasion of the liver at 7 and 17 day post-infection (DPI, d 26 and 35) (Fig. 2). Challenged birds fed diets supplemented with *E*. *faecium* NCIMB 11181 showed lower *C*. *perfringens* numbers in the liver at d 26 compared with the NE-infected birds fed a basal diet. In addition, feeding *E*. *faecium* significantly reduced the number of *C*. *perfringens* cells in the liver compared with the unsupplemented group regardless of challenge (*P* < 0.05).

### Gut lesion scores and histopathological observations

**Figure 3:**
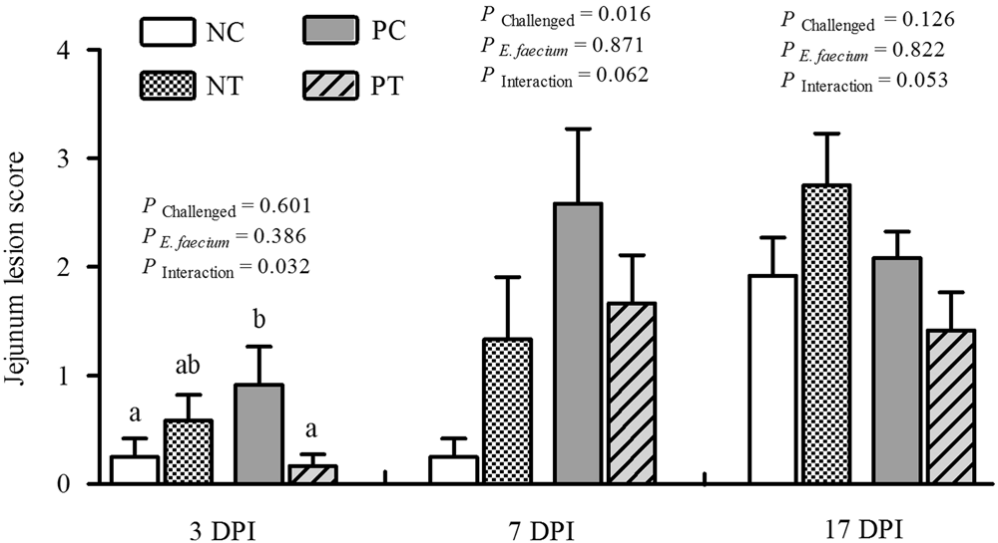
Effect of dietary *E*. *faecium* 11181 on lesion scores in the jejunum of broiler chickens challenged with NE. Values are means (n = 6) with the standard error of the mean (SEM) represented by vertical bars. The lowercase letters on the bars indicate significant differences (*P* < 0.05). NC = Non-NE-infected + no *E. faecium* treatment, NT = non-NE-infected + *E. faecium* treatment, PC = NE-infected + no *E. faecium* treatment, PT = NE-infected + *E. faecium* treatment.

**Figure 4:**
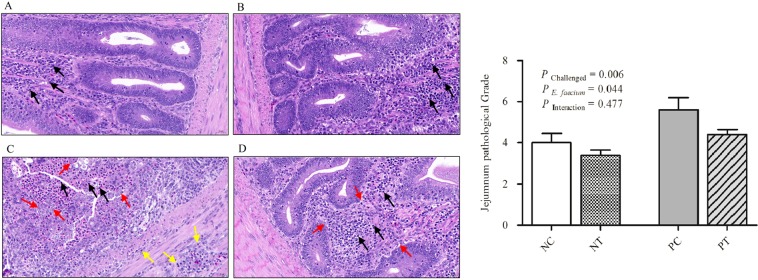
Effect of dietary *E*. *faecium* 11181 on histopathological changes and pathological grade of the jejunum of broiler chickens challenged with NE at d 26 (7 DPI). Histopathological changes in the (**A**) the NC group, (**B**) the NT group, (**C**) the PC group, and (**D**) the PT group. (**E**) Histopathological grade of the jejunum at d 26 (7 DPI). Values are means (n = 6) with SEM represented by vertical bars. Magni*fi*cation = 400×. The black arrow shows inflammatory-cell infiltration, the red arrow shows the necrotic cell clusters with shrunken nucleoli or karyorrhexis and karyolysis, and the yellow arrow shows the connective tissue hyperplasia and inflammatory-cell infiltration in the submucosal and muscle layer.

As shown in Fig. 3, although gut lesion scores were not influenced by *E*. *faecium* supplementation, NE infection caused a significant increase (*P* < 0.05) in gut lesion scores in uninfected birds at 7 DPI (d 26). In addition, there was a notable interactive effect (*P* < 0.05) between *E*. *faecium* supplementation and NE challenge in relation to the jejunum lesion scores at 3 DPI (d 21); NE-infected birds receiving *E*. *faecium*-supplemented diets exhibited a significant score decrease at 3 DPI (*P* < 0.05). Figure 4A–D shows the histopathological observations at 7 DPI, which revealed that the lesions in the NC (birds without NE infection and feed without *E*. *faecium*, Fig. 4A) and NT (birds without NE infection, but feed supplemented with *E*. *faecium*, Fig. 4B) groups were only found in the mucosal layer. The intestinal glands were arranged closely without significant reduction. We observed inflammatory-cell infiltration in the lamina propria, which included plasmocytes and the lymphocytes (black arrows). In the PC group (birds infected with NE, but feed without *E*. *faecium*, Fig. 4C), the lesion was serious and invaded the submucosal and muscle layer. The mucosa was not visible and more necrotic cell clusters were observed with shrunken nucleoli or karyorrhexis and karyolysis (red arrow). In addition, a large amount of inflammatory-cell infiltration was observed including heterophils, granulocytes, and lymphocytes (black arrows). Furthermore, connective tissue hyperplasia and inflammatory-cell infiltration appeared in the submucosal and muscle layer (yellow arrow). The lesion injury in the PT group (birds infected with NE, and feed supplemented with *E*. *faecium*, Fig. 4D) was only in the mucosal layer and did not invade the submucosal and muscle layer. The intestinal glands in the mucosa were loosely arranged and fewer in number (red arrow), and inflammatory-cell infiltration in connective tissue was observed mainly in lymphocytes (black arrow). Histopathological analysis (Fig. 4E) revealed that the NE challenge resulted in an increase in the pathological grade of the jejunum, while NE-induced morphological/structural damage of the intestine and inflammation were attenuated by supplementation with *E*. *faecium* NCIMB 11181.

### Intestinal-cell proliferation and apoptosis

**Figure 5:**
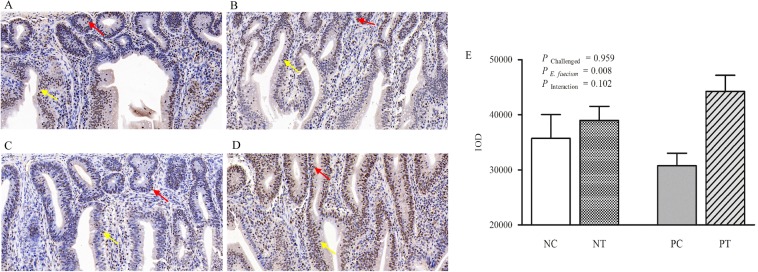
Effect of dietary *E*. *faecium* 11181 on the percentage of PCNA-positive cells in the jejunum of broiler chickens challenged with NE at d 26 (7 DPI). PCNA protein expression was assessed in jejunum tissue by immunohistochemistry using the anti-PCNA antibody in (**A**) the NC group, (**B**) the NT group, (**C**) the PC group, and (**D**) the PT group. The red arrow points out a PCNA-positive cell in the jejunal crypt and the yellow arrow points out a PCNA-positive cell in the jejunal villus. (**E**) Integral optical density (IOD) of PCNA expression. Values are means (n = 6) with SEM represented by vertical bars.  Magnification = 400×.

**Figure 6:**
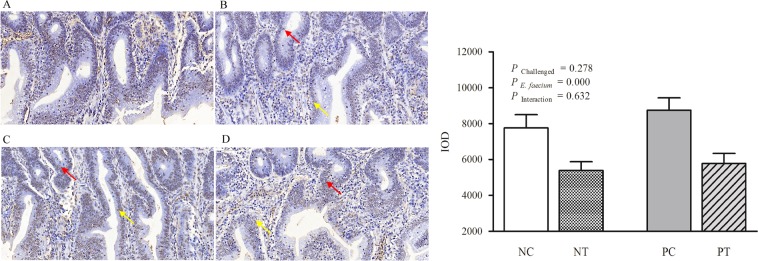
Effect of dietary *E*. *faecium* 11181 on the percentage of TUNEL-positive cells in the jejunum of broiler chickens challenged with NE at d 26 (7 DPI). A TUNEL assay in jejunum sections after 7 days of NE infection in broiler chickens from (**A**) the NC group, (**B**) the NT group, (**C**) the PC group, and (**D**) the PT group. The blue color represents the live cells in the jejunal villus, and the brown color represents the apoptotic cells. The red arrow points out a typical apoptotic cell in the jejunal crypt and the yellow arrow points out a typical apoptotic cell in the jejunal villus. (**E**) Integral optical density (IOD) of TUNEL expression. Values are means (n = 6) with the SEM represented by vertical bars.  Magnification = 400×.

The proliferation (brownish, PCNA-positive cells) and apoptosis (brownish, TUNEL-positive cells) of the epithelial cells at d 26 in the jejunum are shown in Figs 5 and 6, respectively. Although NE infection had no significant effect on cell proliferation and apoptosis in the jejunum compared with non-infected birds, *E*. *faecium* administration resulted in an obvious increase (*P* < 0.05) in the number of PCNA-positive cells (cell proliferation marker) and an obvious reduction (*P* < 0.05) in the number of TUNEL-positive cells (cell apoptosis marker) in the jejunal villus compared with that of untreated birds.

### Cecal microbiome

**Figure 7:**
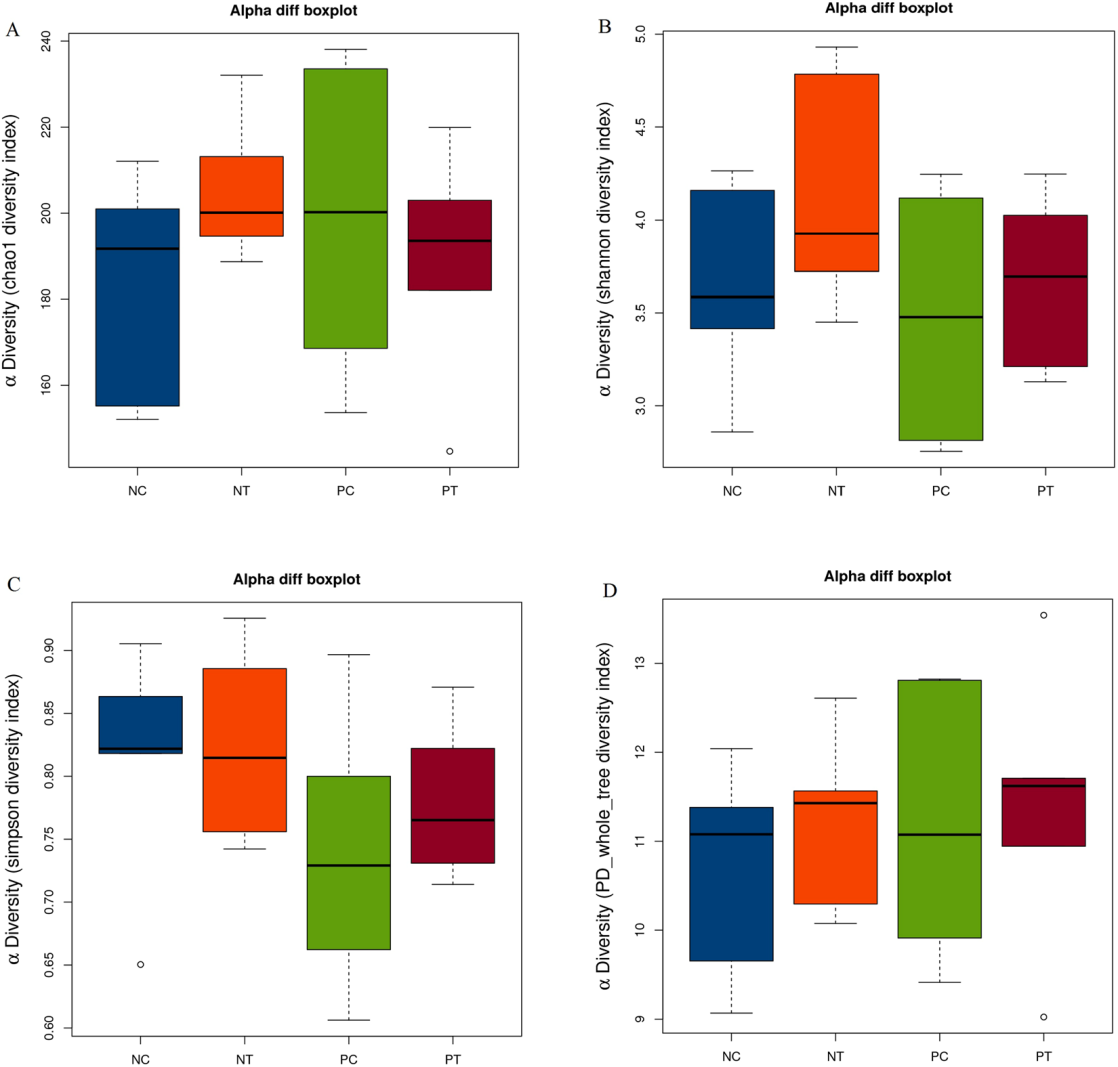
Effect of dietary *E*. *faecium* 11181 on the cecal microbiota α-diversity indices (in cecal feces) of broiler chickens challenged with NE at d 26 (n = 6). (**A**) Chao diversity index. (**B**) Shannon diversity index. (**C**) Simpson diversity index. (**D**) PD whole tree diversity index. NC = Non-NE-infected + no *E. faecium* treatment, NT = non-NE-infected + *E. faecium* treatment, PC = NE-infected + no *E. faecium* treatment, PT = NE-infected + *E. faecium* treatment.

**Figure 8:**
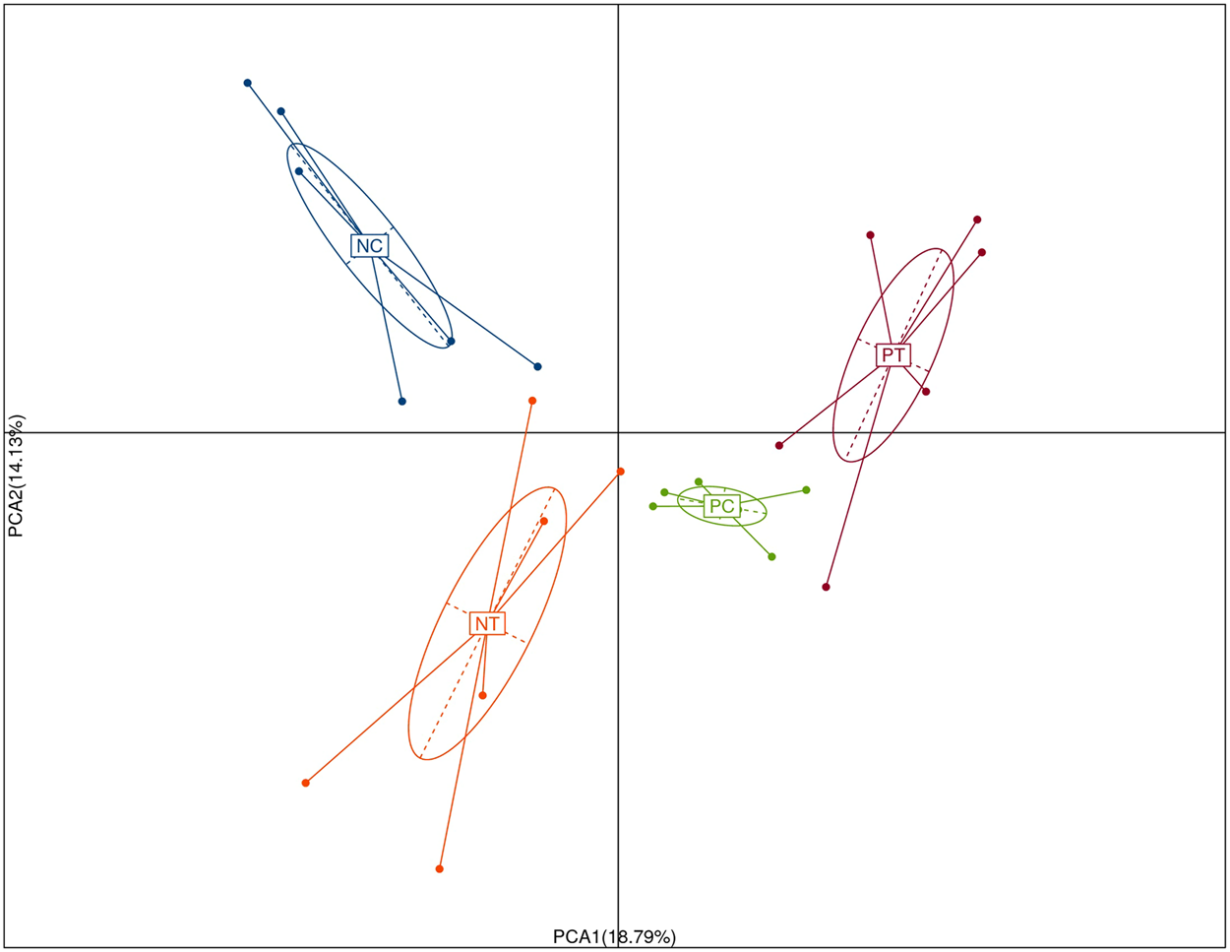
Effects of dietary *E*. *faecium* 11181 on cecal microbiota β-diversity (community similarity) of broilers challenged with NE at d 26 (n = 6). Principal component analysis (PCA) plot of samples in different treatment groups using the abundance information at the genus level. NC = Non-NE-infected + no *E. faecium*treatment, NT = non-NE-infected + *E. faecium* treatment, PC = NE-infected + no *E. faecium* treatment, PT = NE-infected + *E. faecium* treatment.

**Figure 9:**
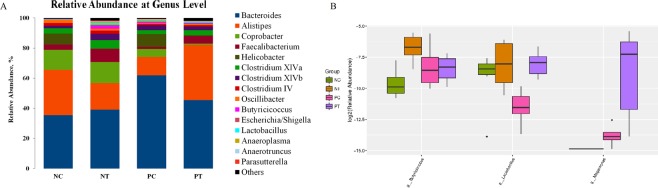
Effect of dietary *E*. *faecium* 11181 on the taxonomic composition of cecal microbiota in broiler chickens challenged with NE at d 26 (n = 6). (**A**) Relative abundance of the top 15 microorganisms at the genus level. (**B**) Different abundances at genus levels. NC = Non-NE-infected + no *E. faecium* treatment, NT = non-NE-infected + *E. faecium* treatment, PC = NE-infected + no *E. faecium* treatment, PT = NE-infected + *E. faecium* treatment.

To characterize the phylogenetic composition of bacterial communities in the cecum, we first performed 16S rRNA gene sequencing analysis and compared the α-diversity and β-diversity of microbiota in the cecum of the different treatment groups. Our results revealed that the α-diversity of the cecal microbiota on d 26 (Table 1 and Fig. 7) was not (*P* > 0.05) influenced by NE infection or probiotic treatment. However, a principal component analysis (PCA) revealed (Fig. 8) a clear difference in microbial community composition among the different groups, indicating significant variability in their microbial profiles. Furthermore, we assessed the taxonomic profiles of the numerically abundant bacteria by analyzing the 16S rRNA gene sequences of the cecum (Fig. 9A and Supplemented Fig. 1). NE infection without *E*. *faecium* treatment resulted in a relative reduction in the proportion of the genus *Lactobacillus* compared with the non-infected and untreated group, while the group fed *E*. *faecium* but without NE infection showed the highest relative abundance (*P* < 0.05) of *Lactobacillus* and *Butyricicoccus* in the cecum compared to the negative control. Compared with the NE-infected group without probiotic treatment, the infected birds fed *E*. *faecium* did not differ in their relative abundance of the genera *Butyricicoccus* but *Lactobacillus* in the cecum (Fig. 9B).

**Table 1:** Effect of dietary *E*. *faecium* on pyrosequencing data and microbiota α-diversity indices in cecal feces of broiler chickens challenged with NE (d 26, 7 DPI).

Groups	Chao 1	Observed Species (ACE)	PD-Whole Tree	Shannon	Simpson
NC	183.97	159.50	10.72	3.64	0.81
NT	204.80	181.33	11.23	4.12	0.82
PC	199.06	174.83	11.18	3.48	0.74
PT	189.47	171.00	11.41	3.67	0.78
*P*- Values	0.62	0.68	0.75	0.49	0.37

NC, neither NE infection nor *E*. *faecium* in the feed; NT, no NE infection but with *E*. *faecium* in the feed; PC, with NE infection but without *E*. *faecium* in the feed; PT, NE infection and *E*. *faecium* in the feed.

### Jejunal TLR signaling pathway immune-related cytokines- and growth factor-associated gene expression

As shown in Tables 2, 3, NE infection significantly upregulated the mRNA levels of the immune-related molecules TLR-2, IL-1β, IL-4, IL-10, IFN-γ, and iNOS and the growth factors TGF-β3 and IGF-2. Conversely, the NE challenge significantly downregulated mRNA expression of the TLR-signal-pathway negative regulator A20 in the jejunum compared with the expression levels in the non-infected groups. Compared with non-supplemented groups, inclusion of *E*. *faecium* to the diet remarkably increased gene expression levels of TLR2, MyD88, NF-κB, IL-4, iNOS, TGF-β3, PI3K, IGF-2, GLP-2, and EGFR; however, the ratio of IFN-γ to IL-4 decreased, and HSP70 protein level was increased as well. In addition, there was a significant interactive effect of NE infection and *E*. *faecium* treatment on MyD88, NF-κB, IL-1β, IL-4, iNOS, PI3K, GLP-2, HSP70 and HSP90 mRNA expression (*P* < 0.05); specifically, NE-infected birds fed with *E*. *faecium* displayed a notable increase in the mRNA levels of these genes.

**Table 2:** Effect of dietary *E*. *faecium* 11181 on mRNA expression of TLR signaling pathway-related genes and downstream cytokines (on d 26, 7 DPI) in the jejunum of broiler chickens challenged with NE.

Dosage (g/T)	Treatment^1^	TLR-2	MyD88	NF-κB	IL-1β	IL-4	IL-8	IL-10	TNFα	IFN-γ	IFN-γ/IL-4	iNOS	Tollip	A20	PI3K
0	−	1.02	1.00^a^	1.00^a^	1.02^a^	1.04^a^	1.09	1.14	1.01	1.05	0.52	1.03^a^	1.02	1.01	1.00^ab^
200	−	1.26	1.33^b^	1.04^ab^	0.95^a^	1.29^a^	0.73	1.13	0.89	1.05	0.37	1.09^a^	1.17	1.01	1.08^ab^
0	+	1.55	1.27^b^	0.89^a^	1.39^a^	1.30^a^	0.68	3.40	0.83	1.59	0.49	1.34^a^	0.95	0.80	0.82^a^
200	+	2.32	1.27^b^	1.22^b^	2.44^b^	3.16^b^	1.30	3.15	0.86	2.13	0.19	2.39^b^	1.04	0.89	1.30^b^
SEM^2^		0.112	0.038	0.034	0.125	0.180	0.132	0.224	0.036	0.090	0.028	0.098	0.031	0.018	0.040
**Main factors**															
Non-challenged		1.14^a^	1.17	1.02	1.06	0.99	1.17	1.11^b^	0.95	1.05^a^	0.45	0.91	1.10	1.01^b^	1.04
Challenged		1.93^b^	1.27	1.06	1.86	1.92	2.23	3.27^a^	0.85	1.86^b^	0.34	0.99	1.00	0.84^a^	1.06
0		1.28^c^	1.14	0.95	1.19	1.21	1.17	2.27	0.92	1.32	0.50^a^	0.89	0.99	0.90	0.91^a^
200		1.79^d^	1.30	1.13	1.74	1.70	2.23	2.14	0.88	1.59	0.28^b^	1.02	1.11	0.95	1.19^b^
**Main factors and Interaction (** ***P-*** **Values)** ^**3**^															
Challenged		0.002	0.184	0.619	0.001	0.008	0.757	0.000	0.169	0.000	0.079	0.001	0.112	0.000	0.788
*E*. *faecium*		0.035	0.049	0.014	0.067	0.008	0.635	0.780	0.533	0.140	0.001	0.011	0.067	0.184	0.003
Challenged × *E*. *faecium*		0.249	0.048	0.042	0.036	0.036	0.076	0.794	0.302	0.155	0.210	0.020	0.652	0.208	0.019

^a,b,c,d^Means in the same column without common superscripts differ significantly (P < 0.05).

^1^Co-challenged with *Eimeria spp.*and *C. perfringens*; -, without NE challenge; + , with NE challenge.

^2^SEM, standard error of the mean.

^3^*P*-value represents the main effect of the diet, the main effect of NE challenge, and the interaction between the dietary treatments and NE challenge.

**Table 3:** Effect of dietary *E*. *faecium* 11181 on the mRNA expression of growth factors, heat-shock proteins, and tight-junction proteins (on d 26, 7 DPI) in the jejunum of broiler chickens challenged with NE.

Dosage (g/T)	Treatment^1^	TGF-β2	TGF-β3	IGF-2	GLP-2	EGFR	HSP60	HSP70	HSP90	MLCK	CLDN-1	CLDN-3	OCLD	ZO-1
0	−	1.02	1.03	1.07	1.02^ab^	1.02	1.03	1.06^a^	1.04^b^	1.03	1.02	1.00^b^	1.01^ab^	1.01
200	−	1.05	1.44	1.15	1.16^bc^	1.12	0.98	1.03^a^	0.86^ab^	0.84	1.16	1.09^b^	1.10^ab^	1.06
0	+	0.88	1.26	1.69	0.74^a^	0.87	1.13	0.97^a^	0.79^a^	1.47	0.88	0.90^b^	1.20^b^	0.79
200	+	1.38	2.21	2.79	1.50^c^	1.12	1.16	2.08^b^	1.07^b^	1.09	1.38	0.51^a^	0.72^a^	0.98
SEM^2^		0.066	0.093	0.14	0.064	0.033	0.050	0.129	0.051	0.078	0.078	0.045	0.064	0.03
Main factors														
Non-challenged		1.03	1.23^a^	1.11^a^	1.09	1.07	1.01	1.05	0.95	0.94^a^	1.13	1.05	1.05	1.03^b^
Challenged		1.13	1.73^b^	2.24^b^	1.12	1.00	1.14	1.53	0.93	1.28^b^	1.43	0.71	0.96	0.89^a^
0		0.95	1.14^c^	1.38^c^	0.88^a^	0.94^a^	1.08	1.02	0.92	1.25	1.06^a^	0.95	1.10	0.90
200		1.21	1.82^d^	1.97^d^	1.33^b^	1.12^b^	1.07	1.55	0.97	0.97	1.50^b^	0.80	0.91	1.02
Main factors and Interaction (*P*- Values)^3^														
Challenged		0.482	0.015	0.001	0.801	0.304	0.182	0.082	0.850	0.043	0.056	0.001	0.456	0.024
*E*. *faecium*		0.056	0.002	0.048	0.003	0.014	0.917	0.052	0.612	0.086	0.007	0.109	0.141	0.068
Challenged × *E*. *faecium*		0.094	0.166	0.087	0.028	0.262	0.659	0.039	0.038	0.542	0.102	0.015	0.038	0.278

^a,b,c,d^Means in the same column without common superscripts differ significantly (P < 0.05).

^1^Co-challenged with *Eimeria spp.* and *C. perfringens*; -, without NE challenge; +, with NE challenge.

^2^SEM, standard error of the mean.

^3^*P*-value represents the main effect of the diet, the main effect of NE challenge, and the interaction between the dietary treatments and NE challenge.

### Jejunal tight-junction gene and protein expression

**Figure 10:**
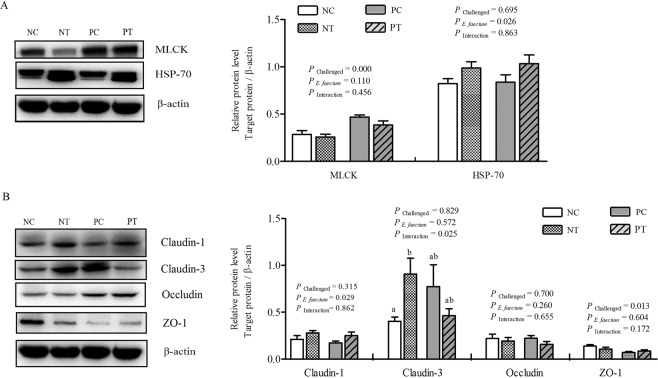
Effect of dietary *E*. *faecium* 11181 on tight-junction protein levels in the jejunum of broiler chickens challenged with NE at d 26. (**A**) MLCK and HSP70 (**B**) Claudin-1, Claudin-3, Occludin and ZO-1. Each result represents the mean value ± SEM (n = 6). The lowercase letters on the bars indicate significant differences (*P* < 0.05). NC = Non-NE-infected + no *E. faecium* treatment, NT = non-NE-infected + *E. faecium* treatment, PC = NE-infected + no *E. faecium* treatment, PT = NE-infected + *E. faecium* treatment.

Reverse transcription (RT)-PCR analysis (Table 3) revealed that NE infection sharply downregulated (*P* < 0.05) the expression levels of claudin-3 (CLDN-3) and zona occludens-1 (ZO-1) genes and upregulated (*P* < 0.05) the myosin light chain kinase (MLCK)-gene mRNA expression in the jejunum compared with the expression in non-infected birds. Compared with the non-supplemented groups, probiotic administration notably upregulated (*P* < 0.05) claudin-1 (CLDN-1) mRNA levels. In addition, a significant cooperative relationship was observed for CLDN-3 and occludin (OCLN) gene expression levels between *E*. *faecium* supplementation and NE infection. NE-induced increases in CLDN-3 and OCLN mRNA levels were alleviated by *E*. *faecium* supplementation. Western Blot (WB) results (Fig. 10) indicated that NE infection resulted in a significant increase in the relative levels of MLCK protein and a reduction in ZO-1 protein levels in the jejunum mucosa compared with the non-challenged groups but no significant difference was observed in the abundance of CLDN-1, CLDN-3, and OCLN protein between the infected and non-infected groups. However, birds fed *E*. *faecium* showed a significant upregulation in CLDN-1 protein levels. A cooperative effect between *E*. *faecium* supplementation and NE infection was noted for CLDN-3 protein expression (*P* < 0.05). The highest CLDN-3 protein levels were observed in the probiotic-treated, non-infected birds (*P* < 0.05), whereas no clear difference was observed in CLDN-3 protein levels among the other three treatment groups.

## Discussion

The subclinical form of *C. perfringens* associated-NE can adversely affect growth performance in poultry^1,6^. Poor growth performance is considered to be primarily caused by coccidia-induced leakage of proteins, including plasma, into the lumen of the small intestine; this condition provides nutrient substrates that facilitate the rapid replication of *C. perfringens* and concomitant damage to the digestive ability of the small intestine^1^. In accordance with previous studies pertaining to growth performance^38,39^, we observed that NE exerted a significant negative influence on BWG of broiler chickens. Conversely, *E. faecium* NCIMB 11181 addition to animal feed is effective in improving growth performance following NE infection. The positive effect of dietary *E. faecium* supplementation in relation to growth performance was also confirmed in other birds^29,37,40^ and pigs^23,41^.

Intestinal lesion scores, histopathological grades, bacterial translocation, and intestinal-cell proliferation and apoptosis indices are important indicators of intestinal health, recovery, and function. In this study, NE infection resulted in a significant increase in gut lesion scores, intestinal-cell apoptosis, bacterial load in the liver, and intestinal histopathological grades. These results, which are consistent with previous studies, indicated that NE can damage gut barrier structure, enhance gut permeability, and induce intestinal inflammation in broiler chickens^6,38,39^. Nevertheless, we showed that NE-induced gut injury and intestinal-cell apoptosis was attenuated and the NE-induced increase of *C. perfringens* in the liver was partially suppressed by *E. faecium* NCIMB 11181 supplementation. Similar effects of probiotics were also reported in previous studies on birds^38,42^. Thus, our findings suggest that pretreatment with probiotic *E. faecium* can protect intestinal epithelial barrier integrity from NE infection by reducing intestinal inflammation and inhibiting apoptosis of intestinal epithelial cells.

The intestinal barrier is regulated by tight-junction proteins (TJPs) that consist of several unique proteins including the junction adhesion molecule, the transmembrane protein occludin, members of the claudin family, and linker proteins such as the zonula occludin protein family^43^. This mechanical barrier plays an important role in the absorption of nutrients, electrolytes, and water, as well as the maintenance of intestinal-barrier integrity and function and the protection of the gut from enteric pathogen invasion^44,45^. Intestinal TJ barrier disruption not only increases membrane permeability to luminal antigens and bacterial translocation (leading to endogenous infection, sustained inflammation and tissue damage) but also reduces the absorption of nutrients^7,45–47^. In this study, NE infection significantly downregulated CLDN-3 and ZO-1 mRNA levels and ZO-1 protein content but upregulated MLCK mRNA expression and protein levels in the jejunum compared with the non-infected birds. Several studies in birds and humans have demonstrated that enterotoxin produced by *C*. *perfringens* disrupted the intestinal TJP complex, altered gut-barrier function, and led to intestinal epithelial cell apoptosis and death^7,8^. Additionally, the intestinal MLCK pathway is involved in both the degradation or distribution of TJs and intestinal permeability^8,48^. Thus, the occurrence of these phenomena suggests that NE infection disrupts multiple intestinal TJPs and compromises the structural integrity of the gut barrier, thereby leading to bacterial translocation into the liver due to the increased gut permeability. Pretreatment with *E*. *faecium* notably upregulated CLDN-1 mRNA levels and protein content in the jejunum mucosa of birds, regardless of NE infection. Furthermore, non-infected birds fed *E*. *faecium* showed higher CLDN-3 protein levels compared with the NE-infected birds. CLDN-1 is a member of the multiple-spanning, transmembrane, claudin protein family, which comprises of more than 20 members and has been shown to play an important role in barrier formation and paracellular selectivity in various tissues^45^. Thus, our results suggest that *E. faecium* addition protected intestinal barrier function against intestinal pathogens possibly via the upregulation of CLDN-1 protein levels. Enhanced tight junctions following *E. faecium* supplementation resulted in reduced gut permeability and reduced pathogen invasion. In accordance with our findings, several other studies have shown that probiotic *E. faecium* reinforced epithelial barrier function in pigs and mice with intestinal inflammation^19,49,50^ and prevented Enterotoxigenic *E*. *coli* (*ETEC*)-induced reductions in CLDN-1 mRNA and protein levels in pig cell models^26–28^.

TLR-mediated signaling pathways are involved in regulating intestinal epithelial barrier integrity^51^. Pro-inflammatory cytokines such as TNF-α, IFN-γ, and IL-1β have been reported to increase intestinal permeability and tissue damage via the dysregulation of TJPs^52,53^, while various regulatory peptides including anti-inflammatory cytokines (TGF-β, IL-4 and IL-10), growth factors (EGF, GLP-2 and IGF-2), negative regulators (A20, Tollip and PI3K) of the TLR signaling pathway, and HSPs have been demonstrated to protect intestinal barrier function by regulating tight junction expression and facilitating the repair of damaged gut tissue^54-57^. In order to elucidate the mechanism by which dietary probiotic *E. faecium* NCIMB 11181 supplementation affects intestinal barrier function and health, we further evaluated the changes in the intestinal mucosal toll-like receptors (TLR) and their downstream targets in NE-infected broiler chickens. We found that the NE challenge not only increased the mRNA expression of pro-inflammatory cytokines like TLR-2, IL-1β, IL-4, IFN-γ, and iNOS but also that of the anti-inflammatory cytokine IL-10 in the gut of the infected chickens; these results are similar to previous reports in broilers^58,59^. Moreover, our results revealed that NE infection upregulated the mRNA levels of the growth factors TGF-β3 and IGF-2 while reducing the levels of TLR-signaling-pathway negative regulator A20 in the jejunum compared with the non-infected groups. These results showed that NE infection differentially modulated intestinal immune-related gene expression, resulting in the activation of intestinal immune-inflammatory responses. Compared with non-supplemented groups, inclusion of *E*. *faecium* in the diet remarkably increased the gene expression levels of TLR-2, MyD88, NF-κB, iNOS, TGF-β3, PI3K, IGF-2, GLP-2, and EGFR. In accordance with our results, previous studies have demonstrated that *E*. *faecium* affected the expression of intestinal immune-related genes, growth factors, and HSPs in *vivo*^40,60,61^ and *in vitro*^26,27,49,62^. In addition, NE-infected birds fed with *E*. *faecium* displayed a notable increase in the relative gene expression of MyD88, NF-κB, IL-1β, IL-4, iNOS, PI3K, GLP-2, HSP70, and HSP90. Furthermore, our study also found that *E*. *faecium* NCIMB 11181 administration upregulated HSP70 protein expression in the jejunum irrespective of NE infection. Thus, our findings indicated that *E*. *faecium* administration not only supported effective intestinal innate immune-defense responses against pathogen infection by modulation of the TLR signaling pathway but also regulated intestinal immune balance and prevented excessive inflammation by differentially modulating pro-inflammatory and anti-inflammatory cytokines, growth factors, heat shock proteins, and TLR-signaling negative regulators when confronted with an NE challenge. Enhanced intestinal barrier function in NE-infected birds following *E. faecium* pretreatment might be attributable to the associated increase in the expression of TLR-2, IL-4, iNOS, GLP-2, HSP70, HSP90, and PI3K production in the intestinal tract. Improved intestinal barrier function in NE-infected birds given *E. faecium* pretreatment resulted in reduced bacterial translocation to the liver and less systemic inflammation. Taken together, these findings suggest that pretreatment with probiotic *E. faecium* NCIMB 11181 can effectively prevent intestinal inflammation triggered by NE infection, promote wound healing, and enhance intestinal epithelial barrier function. These results are consistent with previous *in vivo* studies in pigs and poultry^19,31,32^ and *in vitro* studies of *E. faecium* in enterobacterial disease^26–28,62^.

To further investigate the mechanism underlying *E. faecium* mitigating NE-induced gut injury, cecal microbiota structure was analyzed by Illumina MiSeq sequencing. In accordance with previous studies in chickens^41,63^, we observed no significant difference in α-diversity following 16S rRNA gene pyrosequencing of the cecal microbiota in NE-challenged birds with or without *E. faecium* supplementation, suggesting that neither probiotic treatment nor NE infection (alone or in combination) vastly modified the diversity of the intestinal microbiota. This finding could be due to inhibition of the proliferation of minor components of the microbiota by *C. perfringens* infection^63^ and inhibition of *C. perfringens* itself by *E*. *faecium*^14,15,34,64^. However, principal component analysis (PCA) showed that β-diversity of the cecal microbiota was, in fact, altered by *E*. *faecium* addition and NE challenge (both alone and together), indicating that these treatments significantly disturbed intestinal bacterial community profiles.

We also observed an increase in the relative abundance of the genera *Lactobacillus* and *Butyricicoccus* in the cecum of unchallenged-birds receiving *E*. *faecium*, suggesting that the inclusion of *E*. *faecium* in the diet of birds promoted the growth of potentially beneficial bacteria. This is in agreement with previous results^29,41,65^. *Lactobacillus* are responsible for higher levels of anti-inflammatory and systemic responses, and for out-competing and exclusively displacing pathogenic bacteria on the mucosal surfaces of the host^66,67^. *Butyricicoccus* spp. can degrade resistant starch or fiber to produce short-chain fatty acids (SCFA), especially butyric acid^68^. Recent evidence suggested that microbiota-derived butyrate was essential for the regulation of the immune response and the maintenance of intestinal epithelium integrity^69,70^. In addition, *Butyricicoccus* has been shown to play an important role in preventing NE infection in broilers^2,71^. Therefore, the evidence supports that pretreatment with *E*. *faecium* effectively prevents disturbances to the cecal microbiome induced by *C. perfringens* infection by enhancing the proliferation of beneficial bacteria and hindering the growth of potential pathogens. This results in a stable gut ecosystem with greater species richness, a phenomenon that possibly accounts for the significant improvement in growth performance and gut health of broilers. Beneficial intestinal microbiota shapes and promotes gut immunity homeostasis^72^. Furthermore, we speculate that *E. faecium* NCIMB 11181 improves gut barrier integrity by modulating the intestinal microbiome. Further fecal-metabolome analyses are required to explore the impact of probiotic *E*. *faecium* NCIMB 11181 on fecal metabolite profiles. These analyses would allow us to establish possible causal links between probiotic metabolites and gut function.

## Conclusion

Pretreating poultry feed with probiotic *E. faecium* NCIMB 11181 confers a significant protective effect against NE-induced gut injury in broiler chickens, possibly by enhancing the expression of intestinal TJP CLDN-1 and HSP70, differentially modulating cytokine expression, and upregulating intestinal gene expression of the TLR negative-regulator PI3K and the growth factor GLP-2, and modulating the intestinal microflora structure. Further work is required to fully validate these specific mechanisms.

## Materials and Methods

### Animal ethics statement

All study procedures were approved by the Animal Care and Use Committee of China Agricultural University and were in accordance with the Guidelines for Experimental Animals established by the Ministry of Science and Technology (Beijing, China). All efforts were made to minimize the suffering of the animals.

### Experimental design, birds, and diets

A 2 × 2 factorial arrangement was employed in a completely randomized design to investigate the effects of two levels of *E*. *faecium* supplementation (0 or 2 × 10^8^ CFU/kg of feed, the dosage of *E*. *faecium* was based on the recommendation of its producer and our previous study) and two levels of NE (NE-challenged or unchallenged) on broiler chickens. One-day-old, male AA broiler chicks (n = 180) with similar body weights were obtained from a commercial hatchery (Beijing Arbor Acres Poultry Breeding Company). All the birds were weighed and randomly assigned to one of the four experimental groups. Each group had three replicate pens with 15 birds per pen. The treatment groups were as follows: (i) negative control group (birds without NE infection and feed without *E*. *faecium*, **NC**), (ii) *E*. *faecium*-treated group (birds without NE infection, but feed supplemented with *E. faecium*, NT), (iii) NE-infected control group (birds infected with NE, but feed without *E*. *faecium*, **PC**), and (iv) the probiotic-treated and NE-infected group (birds infected with NE, and feed supplemented with *E*. *faecium*, **PT**). Subclinical NE was induced in broiler birds as previously described^38^. The NE-challenged birds were restricted to isolated compartments of the pen to avoid cross-infection. An antibiotic-free, commercial, basic diet was prepared according to the National Research Council (NRC, 1994) requirements for starter (d 1–21) and grower (d 22–42) periods. The composition of the basal feed and associated nutrient levels are presented in Table 4. The probiotic-treated feed included 200 mg *E*. *faecium* per kg of feed (NCIMB11181, viable count ≥1 × 10^9^ CFU/g, obtained from Probiotics International Ltd. Co., UK). The purchased *E*. *faecium* microcapsules are obtained by a sequence of liquid fermentation, filtration, micro-encapsulation, and quick freezing. To ensure the homogeneity of the additives, approximately 5 kg of the basal feed was thoroughly mixed with the additive in a plastic bucket. To ensure that the probiotic dosages were performed correctly, samples of the *E*. *faecium*-treated feed were taken, serial dilutions were made, cultures were grown in sodium azide-crystal violet-esculin agar (CM 1507, Beijing Land Bridge Technology Co., LTD) at 37*°*C for 24 h under an anaerobic environment, and the number of *E. faecium* bacteria was counted using the spread-plate counting method. The chicks were reared on net-floor cages in a closed and ventilated enclosure. Each pen had a floor space of 11,200 (160 × 70) cm^2^ and was equipped with a separate feeding trough and nipple drinkers. In accordance with the AA Broiler Management Guide, all the birds received continuous light for the first 24 h and were then maintained under a 23-h light/1-h dark cycle for the remainder of the study. The temperature in the pen was maintained at 33–34 °C for the first three days and then gradually decreased by 2 °C per week until a final temperature of 22–24 °C was achieved. The relative humidity was kept at 60–70% in the first week and at 50–60% thereafter. All the birds were allowed *ad libitum* access to feed and water throughout the study.

**Table 4:** Composition and nutrient levels of the basal diet.

Ingredient	1 to 21 days	22 to 35 days
Composition (%, unless otherwise noted)		
Corn (7.8% CP)	54.65	60.72
Soybean meal (46% CP)	37.40	32.00
Soybean oil	3.52	3.28
Limestone-calcium carbonate	1.11	1.15
Calcium hydrogen phosphate	2.10	1.63
Sodium chloride	0.30	0.30
DL-Methionine (98%)	0.20	0.20
L-Lysine HCL (98%)	0.23	0.23
Vitamin premix^a^	0.03	0.03
Mineral premix^b^	0.20	0.20
Choline chloride (50%)	0.26	0.26
Total	100.00	100.00
Calculated Nutrient levels^c^		
Metabolizable energy (MJ/kg)	12.33	12.54
Crude protein (%)	21.00	19.11
Calcium (%)	1.00	0.90
Available phosphorus (%)	0.48	0.40
Lysine (%)	1.15	1.04
Methionine (%)	0.50	0.41

^a^Vitamin premix provided per kg of complete diet: vitamin A (retinylacetate), 9500 IU; vitamin D_3_ (cholecalciferol), 2500 IU; vitamin E (DL-a-tocopherol acetate), 30 IU; vitamin K_3_ (menadione sodium bisulfate), 2.65 mg; vitamin B_12_ (cyanocobalamin), 0.025 mg; biotin, 0.30 mg; folic acid, 1.25 mg; nicotinic acid, 50 mg; d-pantothenic acid, 12 mg; pyridoxine hydrochloride, 6.0 mg; riboflavin, 6.5 mg; thiamine mononitrate, 3.0 mg.

^b^Mineral premix provided per kg of complete diet: iron, 80 mg; copper, 8 mg; manganese, 100 mg; zinc, 80 mg; iodine, 0.35 mg; selenium, 0.15 mg.

^c^Calculated value based on the analysis of experimental diets.

### Growth performance

All broilers were individually weighed, and body weight (BW) and average body weight gain (ABWG) were measured at different experimental periods (at d 13–21, d 22–26, d 27–35, and d 21–26). The death rate was calculated during two periods: d 1–12 and d 13–35.

### Intestinal NE lesion scoring and sample collection

On 3, 7 and 14 d post-*C. perfringens* infection (at 21, 26 and 35 days of age), two bird was randomly selected from each replicate pen, weighed, and euthanized by cervical dislocation. The jejunum was collected and scored for NE gut lesions on a scale of 0 (none) to 4 (high) by three independent observers who were blinded to the experimental design, as previously described^73^. On 7 DPI, 1-cm-long samples of the jejunum (between Meckel’s diverticulum and the proximal end of the jejunum), were snap-frozen in liquid nitrogen and stored at *−*80 °C for subsequent mRNA and protein analysis. In addition, ~2-cm-long samples of the jejunum were washed with PBS and then fixed in 4% paraformaldehyde solution for subsequent histopathological observation and immune-histochemical examination. Approximately 3 g of the digesta from the cecum were collected in sterile tubes and immediately frozen at -80 °C for microbial DNA analysis. Liver tissue was aseptically collected and frozen immediately at *-*40 °C for bacterial translocation (BT) analysis.

### Histological and histopathological examination

The fixed jejunum-tissue samples were dehydrated in a tissue processor (Leica Microsystems K. K., Tokyo, Japan) and embedded in paraffin wax as previously described^38^. The embedded tissue was cut into 5-μm sections using a microtome (Leica Microsystems K. K.) and mounted on polylysine-coated glass slides (Boster Corporation, China). Hematoxylin-Eosin (HE) staining was performed using a routine protocol for histological and histopathological analyses. Histopathological examinations were conducted by light microscopy and findings were imaged and analyzed using a pathological image analysis system (Leica Qwin, Jiangsu, China) with a digital camera (DP72; Olympus). The pathological grade of the jejunum was evaluated by summing the evaluated scores of three factors:

(i) Inflammation (score 0–3): 0 = no inflammatory-cell infiltration; 1 = slight inflammatory-cell infiltration; 2 = moderate inflammatory-cell infiltration; 3 = severe inflammatory-cell infiltration. (ii) Extent of lesions (score 0–3): 0 = No lesion; 1 = Lesion in the mucosal layer; 2 = Lesion in the mucosal layer and submucosa; 3 = Transparent cell wall. (iii) Crypt Damage (score 0–4): 0 = No lesion in crypt; 1 = 1/3 crypt lesion; 2 = 2/3 crypt lesion; 3 = only the epithelial surface was intact; 4 = Crypt and epithelium not visible.

### Proliferating cell nuclear antigen (PCNA) immunohistochemistry analysis

The immunostaining of jejunum-tissue sections was performed following the same procedure as described in previous studies^74^. Briefly, the tissue sections were deparaffinized twice with xylene for 10 min and rehydrated in a graded concentration series of ethanol. A microwave oven (MYA-2270M, Haier, Qindao, China) was used for heat-induced antigen retrieval in citrate buffer solution (pH 6.0) for 20 min (5 min at high power [700 W] and 15 min at low power [116 W]). After cooling the tissue to room temperature for 2–3 h, 3% H_2_O_2_ was used to block endogenous peroxidases. To facilitate blocking of non-specific antibody binding, the tissue sections were incubated with 5% (V/V) bovine serum albumin (BSA) in PBST (PBS, pH = 7.4, 0.1%V/V Tween 20) at 37 °C for 30 min. The tissue sections were then incubated with the mouse anti-PCNA primary antibody (1:500 dilution) (GB11010, Wuhan Servicebio technology Co. Ltd., China) overnight at 4 °C and subsequently incubated at 37 °C for 50 min with the appropriate horseradish peroxidase (HRP)-conjugated secondary antibody (Wuhan Servicebio Technology Co. Ltd., China). All the sections were also immunostained with the chromogenic marker diaminobenzidine (DAB, G1211, Wuhan Servicebio Technology Co. Ltd., China) and counterstained with hematoxylin. Finally, the sections were washed, dried, dehydrated, cleared, and mounted with a coverslip. Serial sections were examined under a light microscope (BH-2; Olympus, Japan) with a digital camera (DP72; Olympus), and fields of view showing different regions of the jejunum tissue were selected and captured in each section. Proliferating cells (brown-yellow, PCNA-positive) were measured in high-power fields selected at random (400 × magnification, 50 × 50 μm) with a computerized image-analysis system (MultiScanBase v. 14.02, Computer Scanning System, Warsaw, Poland). Accumulated integral optical density (IOD) for positive staining in each image was analyzed using the Image-Pro Plus 6.0 software (Media Cybernetics, Inc., MD, USA).

### Terminal deoxynucleotidyl transferase dUTP nick end labeling (TUNEL) assay

The TUNEL assay was carried out according to the manufacturer’s instructions for the Apoptosis Detection Kit (11684817910, Roche, USA). Briefly, the paraffin sections of jejunum tissue were dewaxed with 100% xylene and rehydrated in a graded series of ethanol. Next, the activity of endogenous peroxidases was quenched in 3% H_2_O_2_ with distilled water at 37 °C for 10 min, and the sections were incubated with proteinase K (diluted 1:200 in Tris-buffered saline [TBS]) at 37 °C for 5–10 min in a humid chamber. A labeling mixture including digoxin-dUTP in terminal deoxynucleotidyl transferase (TdT) enzyme buffer was added to the sections and incubated at 37 °C for 2 h. After three continuous washes with TBS for 2 min, the sections were incubated with anti-digoxin-biotin conjugate diluted at 1:100 in blocking reagent for 30 min at 37 °C. The tissue sections were subsequently incubated for 1 h at 37 °C with streptavidin-biotin complex (SABC) diluted at 1:100 in TBS. Labeling was visualized with DAB and the sections were counterstained with hematoxylin. The negative control was performed in an identical manner, except that the TdT enzyme buffer was omitted from the incubation. The IOD of TUNEL-positive cells in the jejunum was assessed by a digital microscope and camera system (Nikon DS-Ri1, Japan). For each section, five fields (400 × magnification, 50 × 50 μm) from each area of the image were analyzed using Image-Pro Plus 6.0 (USA) image analysis software. By selecting ‘color-chosen target’ in the options bar of the morphologic analysis system, all TUNEL-positive cells in the field were marked in color. Finally, the ‘calculating’ option was selected in the options bar to automatically calculate the number of cells and the IOD values.

### Intestinal permeability analysis via bacterial-translocation measurements

The number of *C*. *perfringens* cells in the liver was analyzed using the plate-pouring method as previously described^38^. Bacterial translocation was expressed in colony forming units (log_10_ CFU/gram of tissue).

### DNA extraction and pyrosequencing

Total genomic DNA from cecal samples was extracted using the QIAamp Fast Stool Mini Kit (Qiagen, Hilden, Germany) according to the manufacturer’s instructions. DNA integrity was assessed by agarose gel electrophoresis, and then the genomic DNA was used as a template for PCR amplification. The bacterial 16S rRNA V3–V4 gene region was amplified using the KAPA HiFi Hotstart ReadyMix PCR kit (Kapa Biosystems, USA) and primers F341 and R806 (F341: ACTCCTACGGGRSGCAGCAG, R806: GGACTACVVGGGTATCTAATC). PCR amplification was carried out in a 25-µL reaction system and the amplification conditions were as follows: initial pre-denaturation at 98*°*C for 3 min; 30 cycles of denaturation at 95*°*C for 100 s, renaturation at 50*°*C for 60 s, and elongation at 72*°*C for 2 min; and a final elongation step at 72*°*C for 10 min. The amplification product was assessed by agarose gel electrophoresis (5 µl PCR product, 1.5% agarose gel) at 100 V for 60 min to facilitate size verification. The associated product was subsequently purified using an AxyPrepTM DNA Gel Extraction kit (AXYGEN, USA). Finally, 16S rRNA gene sequencing was performed using the Illumina HiSeq PE250 sequencing platform (Illumina, Santa Clara, CA) at the Realbio Technology Co., Ltd. (Shanghai, China) according to the manufacturer’s instructions.

The raw sequence data obtained from the Illumina HiSeq platform were quality-filtered and demultiplexed using the Quantitative Insights into Microbial Ecology (QIIME) version 1.8.0-dev. First, the sequences were trimmed to eliminate all low-quality sequence reads; sequence reads (of 400–440 nt) with an average quality score of at least 25 were retained by SOAPaligner (v 2.21). USEARCH version 7.1 software was used for the trimmed sequence read clusters and cutoffs (based on 97% similar identity) for operational taxonomic units (OUT), and chimeric sequences were identified and removed using UCHIME. α*-*diversity measures, including the number and evenness of species, the observed OTUs/reads, Good’s coverage, Shannon index, Simpson index and Chao 1 were investigated by MOTHUR v.1.35.0, and the significance of these estimates was determined using a Mann-Whitney U test. In addition, jack-knifed β-diversity was calculated from unweighted and weighted UniFrac distances, and a principal component analysis (PCA) was performed in QIIME. A Kruskal-Wallis test was used to determine the significance of the differences between groups. In addition, MetaStat was used to identify the bacterial taxa differentially represented between groups at genus or higher taxonomy levels.

### Real-time polymerase chain reaction (PCR)

Total RNA was isolated from snap-frozen jejunum tissue samples using the RNAiso Plus Kit (TaKaRa, Dalian, China) according to the manufacturer’s instructions. The quality and quantity of the total RNA were measured with a spectrophotometer (NanoDrop-2000, Thermo Fisher Scientific, Waltham, MA) using the 260:280 nm absorbance ratio. First-strand cDNA was synthesized using a PrimeScript^TM^ RT reagent Kit with gDNA Eraser (Perfect Real Time; Takara Biotechnology Co. Ltd., Dalian, China) according to the manufacturer’s instructions. The cDNA was used to perform quantitative real-time PCR (Applied Biosystems 7500 Fast Real-Time PCR System, USA) for target-gene expression according to the standard protocol^38^. Primer sequences (Table 5) for chicken TLR signal pathway-related genes (TLR2, MyD88, NF-κB, IL-1β, IL-4, IL-8, IL-10, IFN-γ, TNF-α, iNOS, Tollip, A20, PI3K), MLCK, tight-junction protein genes (CLDN-1, CLDN-3, OCLN, ZO-1), growth-factor genes (TGF-β2, TGF-β3, IGF-2, GLP-2, EGFR), heat-shock-protein genes (HSP60, HSP70 and HSP90), and the household gene GAPDH were designed and synthesized by Sango Biotech Co., Ltd (Shanghai, China). The result were showed as 2^−△△CT^.

**Table 5:** Sequences of the oligonucleotide primers used for quantitative real-time PCR for immune-related gene expression^a^.

Gene	Primer sequence 5′→3′	GenBank Accession No.	PCR product (bp)
TLR2	F: GATTGTGGACAACATCATTGACTC	NM_001161650	294
	R: AGAGCTGCTTTCAAGTTTTCCC		
MyD88	F: TGCAAGACCATGAAGAACGA	NM_001030962.3	123
	R: TCACGGCAGCAAGAGAGATT		
NF-kB	F: TGGAGAAGGCTATGCAGCTT	NM_205134.1	117
	R: CATCCTGGACAGCAGTGAGA		
IL-1β	F: TCATCTTCTACCGCCTGGAC	NM_204524.1	149
	R: GTAGGTGGCGATGTTGACCT		
IL-4	F: GTGCCCACGCTGTGCTTAC	NM_001007079.1	82
	R: AGGAAACCTCTCCCTGGATGTC		
IL-8	F: GGCTTGCTAGGGGAAATGA	NM_205498.1	200
	R: AGCTGACTCTGACTAGGAAACTGT		
IL-10	F: CGCTGTCACCGCTTCTTCA	NM_001004414.2	272
	R: TCCCGTTCTCATCCATCTTCTC		
IFN-γ	F: CTTCCTGATGGCGTGAAGA	NM_205149.1	127
	R: GAGGATCCACCAGCTTCTGT		
TNF-α	F: CCCCTACCCTGTCCCACAA	NM_204267.1	67
	R: TGAGTACTGCGGAGGGTTCAT		
iNOS	F: GAACAGCCAGCTCATCCGATA	NM_204961.1	103
	R: CCCAAGCTCAATGCACAACTT		
Tollip	F: AAGGCAGGGTGATGACAAAG	NM_001006471	246
	R: AGGAGGTGGTATTGCCACAG		
A20	F: GACATCGTGCTAACAGCTTGGA	XM_003640919.2	180
	R: AGAAAAGAGGGTATCAGGCACAAC		
PI3K	F: CGGATGTTGCCTTACGGTTGT	NM001004410	162
	R: GTTCTTGTCCTTGAGCCACTGAT		
TGF-β2	F: TCATCACCAGGACAGCGTTA	NM_001031045.3	109
	R: TGTGATGGAGCCATTCATGT		
TGF-β3	F: CATCGAGCTCTTCCAGATCC	NM_205454.1	112
	R: GACATCGAAGGACAGCCACT		
IGF-2	F: TGGCTCTGCTGGAAACCTAC	NM_001030342.2	123
	R: ACTTGGCATGAGATGGCTTC		
GLP-2	F: AAGCTTCCCAGTCTGAACCA	NM_001190165.3	119
	R: ATCCTGAGCTCGTCTGCTGT		
EGFR	F: GGTTGGTCTAGGCATCGGTCT	NM 00205497.2	97
	R: TGGTTCGACAAGCTCCCTCT		
HSP 60	F: GGTGATGCTTGCAGTTGATG	NM_001012916.2	123
	R: TTGCCAATTTCCTGATCTCC		
HSP 70	F: CCACCTACGCAAAGAGGAAG	NM_001030793.2	112
	R: TGAGGTGTTGGGTTCCTTTC		
HSP 90	F: TCCTGTCCTGGCTTTAGTTT	NM0010149164	162
	R: AGGTGGCATCTCCTCGGT		
MLCK	F: TTGACATGGAGGTTGTGGAA	NM_001322361.1	119
	R: GAAGTGACGGGACTCCTTGA		
Claudin-1	F: AAGTGCATGGAGGATGACCA	NM_001013611.2	119
	R: GCCACTCTGTTGCCATACCA		
Claudin-3	F: CCAAGATCACCATCGTCTCC	NM_204202.1	113
	R: CACCAGCGGGTTGTAGAAAT		
Occludin	F: AGTTCGACACCGACCTGAAG	NM_205128.1	124
	R: TCCTGGTATTGAGGGCTGTC		
ZO-1	F: ACAGCTCATCACAGCCTCCT	XM_015278981.1	125
	R: TGAAGGGCTTACAGGAATGG		
GAPDH	F: GGTGAAAGTCGGAGTCAACGG	NM204305	108
	R: CGATGAAGGGATCATTGATGGC		

^a^Primers were designed and synthesized by Sango Biotech (Shanghai) Co., Ltd.

F: forward; R: reverse.

TLR-2, toll like receptor-2; MyD88, myeloid differential protein-88; NF-κB, nuclear factor kappa-light-chain-enhancer of activated B cells; HSP, Heat Shock Protein; MLCK, Myosin Light Chain Kinase; ZO-1, Zonula occludens-1.

### Western blot

Frozen jejunal mucosa was homogenized and lysed in ice-cold lysis buffer containing 50 mmol/L Tris-HCl (pH 7.4), 150 mmol/L NaCl, 1% Nonidet P (NP)−40, 0.1% sodium dodecyl sulfate (SDS), 1.0 mmol/L phenylmethylsulfonyl fluoride (PMSF), 1.0 mmol/L sodium orthovanadate (Na_3_VO_4_), 1.0 mmol/L sodium fluoride (NaF), and a protease and phosphatase inhibitor cocktail (P1045, Beyotime Biotechnology, Co., Ltd., Beijing). The resultant cell lysate was centrifuged at 12,000 × *g* for 15 min at 4 °C to remove cellular debris. Protein concentrations were determined using a Pierce™ bicinchoninic acid (BCA) protein assay kit (CW0014, CWBIO Ltd., Beijing). Equal amounts of protein (30 μg) were separated on 8%, 10%, or 12% SDS-polyacrylamide gels (Tris-glycine-SDS-polyacrylamide gel electrophoresis), and transferred to a methanol-presoaked polyvinylidene di*fl*uoride (PVDF) membrane (IPVH000101, Millipore, USA). The membranes were inoculated with 5% skimmed-milk solution in TBS containing 0.05% Tween-20 (TBST blocking solution) at 37 °C for 1 h and were then incubated with primary antibodies overnight at 4 °C. The primary antibodies were anti-MLCK (M7905, Sigma-Aldrich, USA), anti-ZO-1 (61–7300, Invitrogen Corporation, Camarillo, USA), polyclonal rabbit anti-chicken CLDN-1, CLDN-3, and OCLN (antibodies against recombinant chicken CLDN-1, CLDN-3, and OCLN proteins were prepared in our laboratory and patents are currently being authorized). The blots were stripped and incubated with anti-β-actin antibody (A1978, Sigma-Aldrich, USA) to demonstrate equal loading. After incubation at room temperature for 3 h with HRP-conjugated goat anti-rabbit IgG (A0208) or goat anti-mouse IgG antibodies (A0216, Beyotime Biotechnology, Co., Ltd., Beijing), the chemiluminescence signal was detected using the ECL-Plus chemiluminescent kit (Hua Xing Bo Chuang Biotechnology Center, Beijing) and a luminescence imager (Tanon 5200, Tanon Science & Technology Co., Ltd., Shanghai). Quantification of band density was conducted using Image J software.

### Statistical analysis

For the normally distributed data (growth performance, gut lesion scores, liver *C*. *perfringens*, intestinal histopathological scores, intestinal PCNA-positive and TUNEL-positive cell numbers, relative mRNA expression, protein expression), the main effect and interaction effects were analyzed using the general linear model (GLM) procedure in SPSS 22.0 (SPSS Inc, Chicago, Illinois, USA). The results were expressed as treatment means with their pooled SEM. The one-way ANOVA and multiple comparisons were performed when interactive effects differed significantly. α-diversity and β-diversity were analyzed using the Mann-Whitney U test. The relative abundance of microorganisms obtained from 16S rRNA sequencing was analyzed using the Kruskal-Wallis test to compare the difference between two groups or all four treatments. *P* < 0.05 was considered significant.

## Supplementary information

Supplementary Figure 1. Effect of dietary *E. faecium* 11181 supplementation on taxonomic composition of cecum microbiota relative abundance in broiler chickens challenged with NE (n=6)

Supplementary information Figure 2. Full length blots of all Western blots
