# Supplementary material for: Pretreatment with probiotic Enterococcus faecium NCIMB 11181 ameliorates necrotic enteritis-induced intestinal barrier injury in broiler chickens
Source: Sci Rep. 2019 Jul 16;9:10256. doi: 10.1038/s41598-019-46578-x (PMC6635415; doi:10.1038/s41598-019-46578-x)

Pretreatment with probiotic *Enterococcus faecium* NCIMB 11181 ameliorates necrotic enteritis-induced intestinal barrier injury in broiler chickens

Yuanyuan Wu [1], Wenrui Zhen [1], Yanqiang Geng [1], Zhong Wang [1]*, Yuming Guo [1]

[1]State Key Laboratory of Animal Nutrition, College of Animal Science and Technology, China Agricultural University, Beijing, China.

[*] Corresponding author:
Zhong Wang
Department of Animal Science and Technology
China Agricultural University
No. 2 Yuan Ming Yuan Western Road, Hai Dian District,
Beijing 100193, P. R. China
Phone: +86-10-62732712. Fax:+ 86-10-62732712
E-mail address: wangzh@cau.edu.cn

Key words: Chicken, *Enterococcus faecium*, Necrotic enteritis, Gut health

Supplementary information Figure 1. Effect of dietary *E. faecium* 11181 supplementation on taxonomic composition of cecum microbiota relative abundance in broiler chickens challenged with NE (n=6). A, Relative abundance of top 7 microorganisms at phylum level. B, Relative abundance of top 15 microorganisms at class level. C, Relative abundance of top 15 microorganisms at order level. D, Relative abundance of top 15 microorganisms at family level.

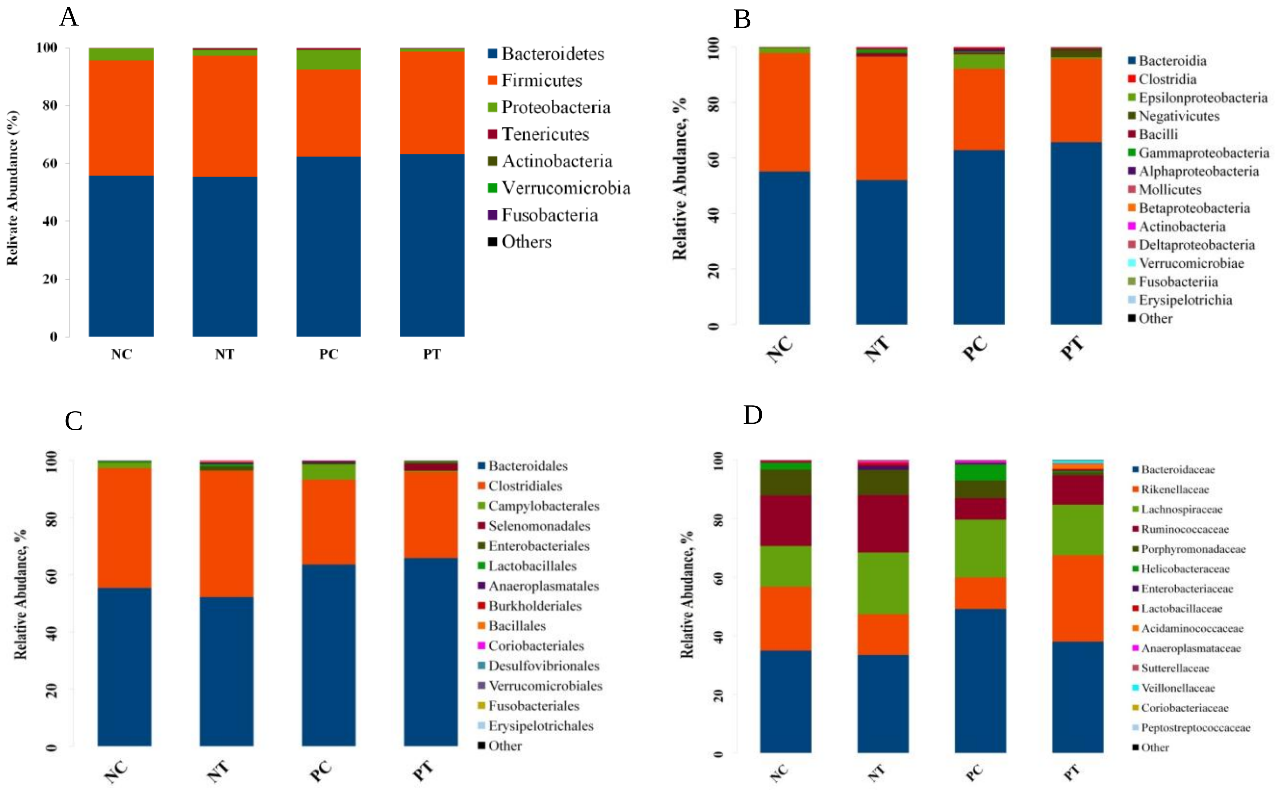

Supplement: Supplementary file 1 — Supplementary Figure 1. Effect of dietary E. faecium 11181 supplementation on taxonomic composition of cecum microbiota relative abundance in broiler chickens challenged with NE (n=6) [file 41598_2019_46578_MOESM1_ESM.pdf]
