# Supplementary material for: Pretreatment with probiotic Enterococcus faecium NCIMB 11181 ameliorates necrotic enteritis-induced intestinal barrier injury in broiler chickens
Source: Sci Rep. 2019 Jul 16;9:10256. doi: 10.1038/s41598-019-46578-x (PMC6635415; doi:10.1038/s41598-019-46578-x)

Pretreatment with probiotic *Enterococcus faecium* NCIMB 11181 ameliorates necrotic enteritis-induced intestinal barrier injury in broiler chickens

Yuanyuan Wu [1], Wenrui Zhen [1], Yanqiang Geng [1], Zhong Wang [1]*, Yuming Guo [1]

[1]State Key Laboratory of Animal Nutrition, College of Animal Science and Technology, China Agricultural University, Beijing, China.

[*] Corresponding author:
Zhong Wang
Department of Animal Science and Technology
China Agricultural University
No. 2 Yuan Ming Yuan Western Road, Hai Dian District,
Beijing 100193, P. R. China
Phone: +86-10-62732712. Fax:+ 86-10-62732712
E-mail address: wangzh@cau.edu.cn

Key words: Chicken, *Enterococcus faecium*, Necrotic enteritis, Gut health

Supplementary information Figure 2. Full length blots of all Western blots.

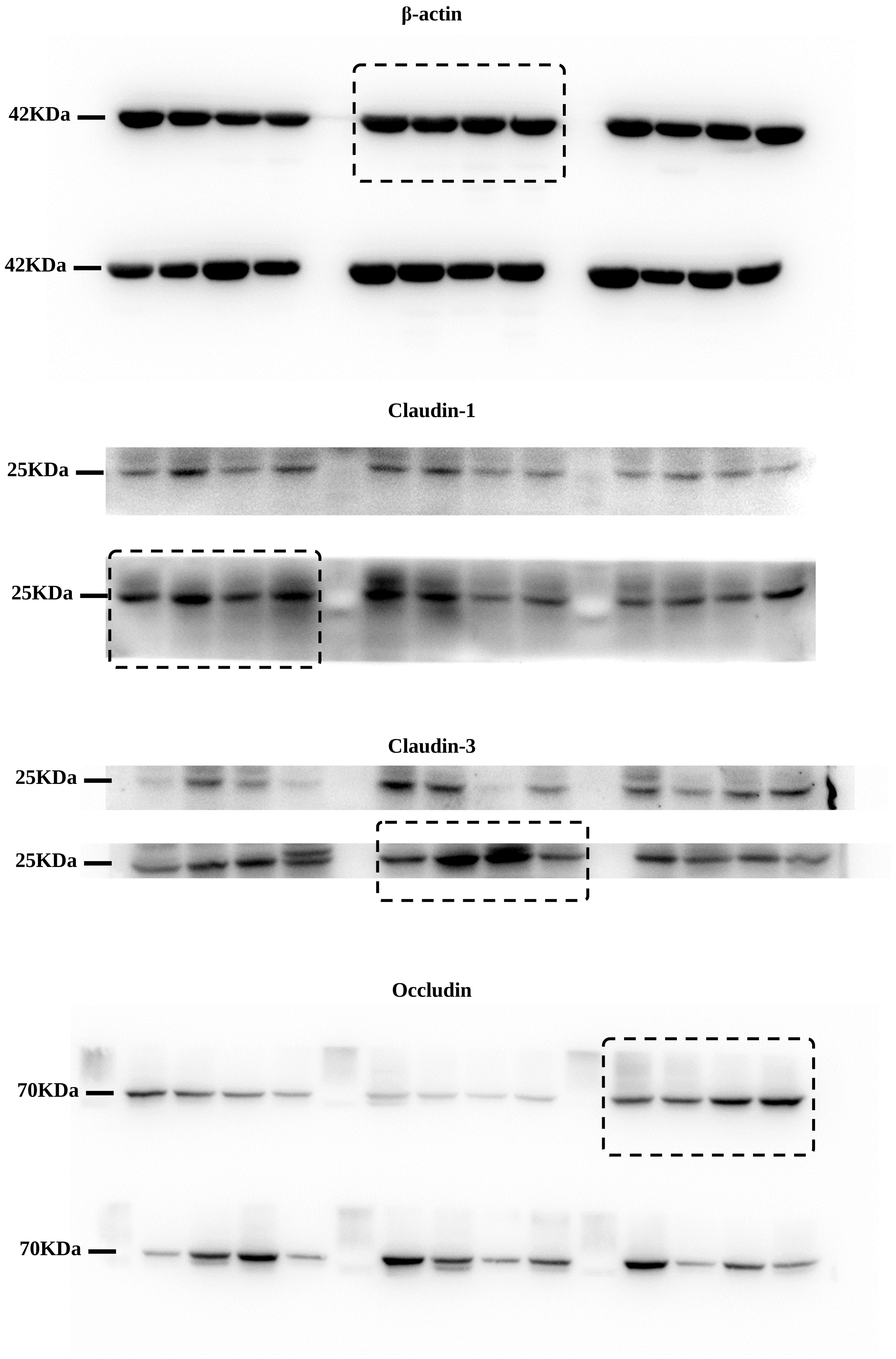

ZO-1

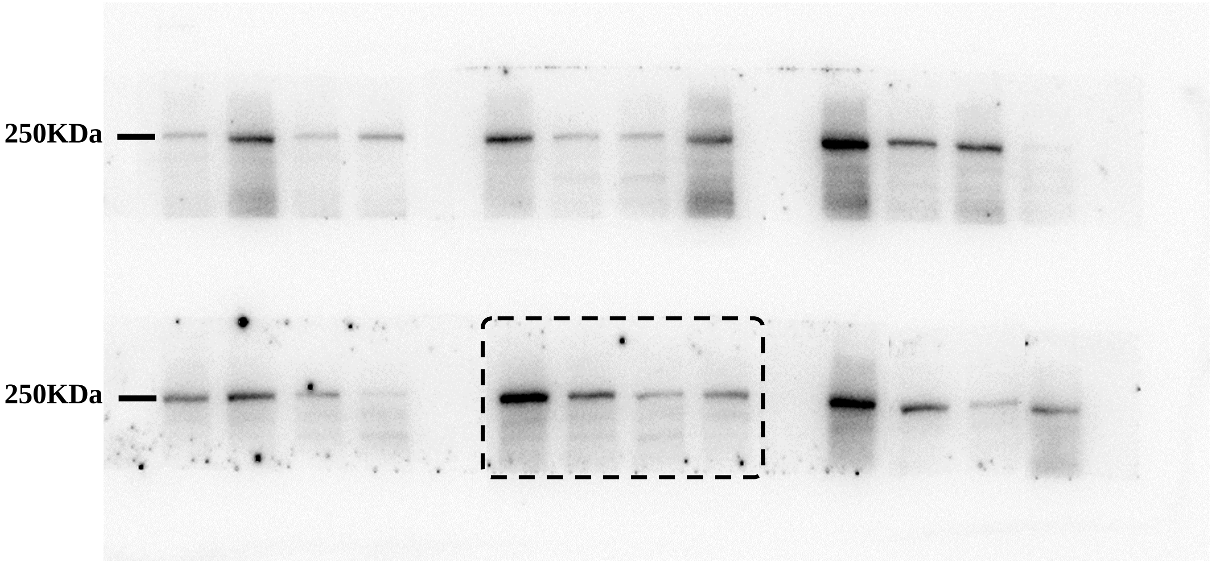

MLCK

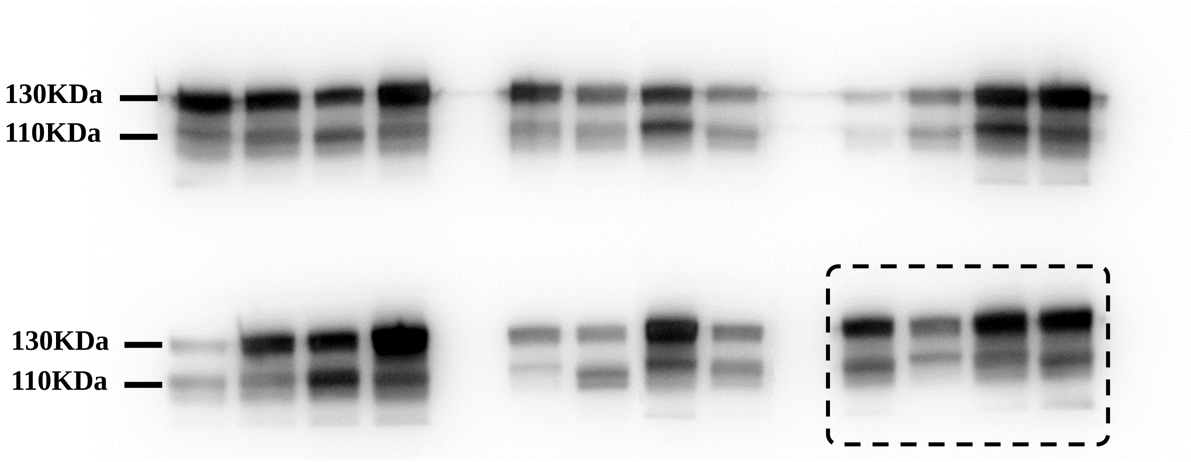

HSP-70

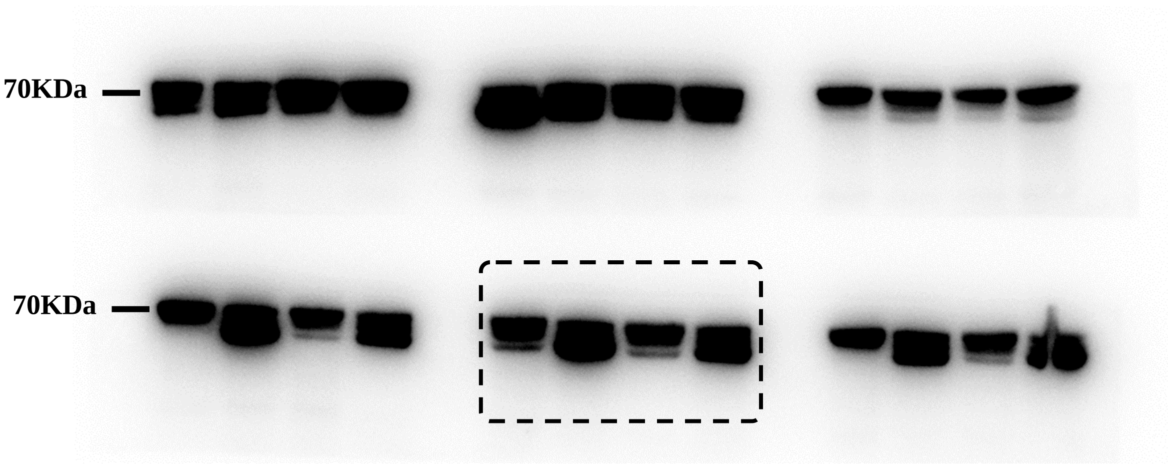

Supplement: Supplementary file 2 — Supplementary information Figure 2. Full length blots of all Western blots [file 41598_2019_46578_MOESM2_ESM.pdf]
